# Possible surface protein markers for breast cancer.

**DOI:** 10.1038/bjc.1981.19

**Published:** 1981-01

**Authors:** M. Wildridge, G. V. Sherbet

## Abstract

**Images:**


*Br. J. Cancer* (1981) **43**, 118

***Short Communication***

### POSSIBLE SURFACE PROTEIN MARKERS FOR BREAST CANCER*

M. WILDRIDGE† AND G. V. SHERBET

*From the Cancer Research Unit, University of Newcastle upon Tyne, Royal Victoria Infirmary, Newcastle upon Tyne NE1 4LP*

Received 6 June 1980 Accepted 29 September 1980

THE IMPORTANCE of tumour markers for the detection of malignancies, and for determining the extent and the clinical course of the disease, has prompted much investigation into the establishment of possible biochemical markers for breast cancer. Although tumour-associated products such as the carcinoembryonic antigen (Wang *et al.*, 1975; Tormey *et al.*, 1977; Cove *et al.*, 1979) and chorionic gonadotropin (Tormey *et al.*, 1976) among others have been suggested as possible candidates, no specific markers have been found for breast cancer.

There is considerable evidence that neoplastic transformation is accompanied by a variety of alterations at the cell surface, particularly in the glycoprotein components (see Hynes, 1979; Sherbet, 1978 for review). Recent work has indicated that a common marker for neoplastic transformation might exist (Price & Stoddart, 1976; Bramwell & Harris, 1978; Bowen & Kulatilake, 1979). The establishment of a marker associated with malignant behaviour of tumours (*i.e.* their ability to invade and form distant metastatic deposits) may be of even greater importance, not only in the understanding of the biology of the tumour but also in the clinical management of patients. Differences in metastatic ability of experimental tumours have recently been shown to be accompanied by changes in the surface components (Brunson *et al.*, 1978; Yogeeswaran *et al.*, 1978; Turner *et al.*, 1981). We describe here a decreased expression of a protein component of mol. wt ~265 K, and an increased occurrence of a 63K component on the surface of cells derived from carcinomas of the breast.

*Tumour-cell cultures.*—Tumour specimens were collected within 30 min after excision from the patients and used to initiate cell cultures as described by Sherbet & Lakshmi (1974). The tumour tissue was dissected free of fat, capsular and necrotic material. The remaining tumour tissue was washed in saline, finely chopped with scissors and No. 22 (Swann-Morton) flat scalpel blades, and placed in 25cm$^2$ culture flasks (Nunc-Flow Labs) with 10 ml of growth medium. The growth medium consisted of Eagle's minimum essential medium containing 20mM Hepes buffer and supplemented with 10% foetal bovine serum, 10% heat-inactivated horse serum, 0·03% $NaHCO_3$, 0·36mM glutamine and antibiotics (0·025% streptomycin sulphate, 500 u/ml penicillin G and 60 u/ml Mycostatin). The flasks were equilibrated with 5% $CO_2$ in air. The explants of tumour tissue were allowed to settle and adhere to the substratum at 37°C. Epithelial cells grow out radially from the explants. At this stage the adherent pieces were detached by gentle shaking and the epithelial cell cultures allowed to grow at 37°C. The cells were subcultured at confluence as follows: they were harvested with the aid

* Part of a thesis to be submitted by M.W. for examination for the Fellowship of the Institute of Medical Laboratory Sciences.

† Present address: Pilgrim Hospital, Boston, Lincs.

Correspondence to Dr G. V. Sherbet.

of 0·25% trypsin and 0·02% EDTA in phosphate-buffered saline without calcium and magnesium, washed twice in growth medium, and $10^6$ viable cells suspended in 25 ml growth medium were inoculated into 75cm$^2$ culture flasks and incubated at 37°C.

Cell cultures at early passages were used in the investigation. In all cultures, epithelial cells predominated, being identifiable by their morphology and tendency to form coherent sheets (Fig. 1). The use of early passages ensured that only small numbers of fibroblasts or their precursors were present among the epithelial cells.

*Radioiodination of monolayer cultures.*—Subconfluent monolayer cultures were used for the radioiodination of surface proteins. The cell monolayer was incubated in phosphate-buffered saline (PBS) pH 7·2 for 30 min at 37°C. This was followed by a final rinse in PBS to wash

FIG. 1.—Morphology of a carcinoma in tissue culture (× ~75).

off adherent serum proteins. This procedure would be expected to remove the majority of adherent serum protein, but it is possible that some strongly adherent proteins may have remained absorbed to the cell surface. On the other hand, certain weakly bound peripheral proteins may have been removed, although this would seem unlikely.

The cell-surface proteins were then labelled by lactoperoxidase-catalysed radioiodination (Hynes, 1973). To each culture flask 2 ml of PBS containing 5mM glucose was added. Carrier-free $Na^{125}I$ (Radiochemical Centre, Amersham) and glucose oxidase (Boehringer) and lactoperoxidase (Calbiochem) were used at 1 mCi, 2·5 $\mu$g and 1 iu respectively per flask. The constituents were mixed gently and the flasks were left at room temperature for 10 min, with occasional gentle shaking. The reaction was terminated by the addition of 5 ml of phosphate-buffered iodine (PBI) containing 0·137M NaI and 2mM phenyl methyl sulphonyl fluoride (PMSF) (proteinase inhibitor). The iodinated monolayer was washed ×3 in PBI–PMSF solution. The cells were scraped off with a polypropylene "policeman" and solubilized in 0·3 ml of 0·01M sodium phosphate buffer (pH 7·0) containing 1% (w/v) sodium dodecyl sulphate, 1% (w/v) mercaptoethanol and 2mM PMSF by incubation for 10 min in a boiling water bath. To each extract was added 0·1 g sucrose. The extracts were stored at −20°C.

Thirty-$\mu$l aliquots of extracts of the labelled material were separated by electrophoresis in 6·0% (w/v) cylindrical polyacrylamide gels (4 mm × 8 cm) containing 200mM sodium phosphate buffer (pH 7·2), 0·2% (w/v) SDS and 0·05% bromophenol blue (Guy *et al.*, 1977). Six replicate gels were run of each labelled extract. After electrophoresis, 1mm-thick slices of the gels were prepared. The radioactive content of each slice was counted in a Nuclear Enterprises 1600 gamma counter and expressed as a percentage of total activity recovered. Standard mol.-wt markers

FIG. 2.—Electrophoretic patterns of radio-iodinated surface proteins of a fibrocystic hyperplasia (top), fibroadenoma (middle) and a carcinoma (bottom).

(B.D.H.) in the range of 280,000–56,000 daltons were used in electrophoretic analysis and for the estimation of the mol. wt of the labelled proteins.

Fig. 2 gives typical electrophoretic patterns of iodinated surface proteins of cells derived from fibrocystic hyperplasias, fibroadenomas and histologically proven carcinomas of the breast. A number of differences were discernible in the patterns, but the most salient ones related to the occurrence of two components of mol. wts ~265 K and 233 K. The former occurred in both fibrocystic hyperplasias and in 4/5 fibroadenomas examined. The

TABLE I.—*Cell-surface proteins of breast tumours*

| Patient | Tumour type | Major components 265 K | 233 K | 145 K | 63 K |
|---|---|---|---|---|---|
| EBA | Fibrocystic | + | | + | + |
| VHG | hyperplasia | + | | + | |
| MAT | Fibroadenoma | ? | | + | |
| AFH | | + | | + | + |
| JFE | | + | | + | |
| MEA | | + | | + | |
| WRA | | + | | ? | |
| MCP | Carcinoma | | ? | + | + |
| HOR | | | + | + | + |
| AME | | | + | + | + |
| BAS | | | ? | + | + |
| MCF | | | + | ? | ? |

The + sign indicates the presence of a well-defined peak of radioiodine incorporation.

233K component occurred as a defined peak in 3/5 carcinomas (Table I) but the difference in radioactive incorporation between fibroadenomas and the carcinoma group was not statistically significant. In Table II the ratios of incorporation of radioiodine with the 265K and 233K components are given. These ratios are higher in the fibroadenoma group than in the carcinoma group. A statistical analysis using the non-parametric statistical technique of Mann and Whitney described by Campbell (1967) revealed that the ratios of the fibroadenoma group differed stochastically from those of the carcinomas ($P \sim 0{\cdot}016$). It may be concluded, therefore, that the 265K peak was less in the carcinomas than the fibroadenomas.

A third feature which seemed to distinguish between the benign conditions and the carcinomas was the incorporation of the label into components of average mol. wt 63 K. The proportion of radioactivity associated with the 63K components was found to be higher in the carcinomas than in the fibroadenomas (Table II). This difference also was statistically significant ($P < 0{\cdot}03$) in the Mann–Whitney test. Minor differences were found in the proportion of radioactivity associated with the 145K protein, but this component was found to occur in

TABLE II.—*Distribution of radioactivity associated with cell-surface components of fibroadenomas and carcinomas of the breast*

| Patients | Tumour type | Mean ct/min total recovered per gel ($\times 10^{-3}$) | % Radioactivity 265 K | % Radioactivity 233 K | 265 K/ 233 K | % Radioactivity 145 K | % Radioactivity 63 K |
|---|---|---|---|---|---|---|---|
| MAT | Fibroadenoma | 26·3 | 5·60 ± 0·9 | 2·80 ± 0·8 | 2·0 | 6·57 ± 1·3 | 17·76 ± 0·7 |
| MEA | | 27·5 | 10·07 ± 0·5 | 1·74 ± 0·8 | 5·79 | 5·56 ± 0·5 | 16·26 ± 0·4 |
| JFE | | 47·5 | 11·92 ± 1·8 | 2·38 ± 0·9 | 5·01 | 6·67 ± 0·4 | 12·70 ± 1·5 |
| WRA | | 34·9 | 6·77 ± 1·4 | 2·22 ± 0·9 | 3·05 | 6·21 ± 0·8 | 16·92 ± 4·0 |
| AFH | | 17·0 | 11·97 ± 1·5 | 2·27 ± 0·9 | 5·27 | 5·79 ± 0·8 | 12·62 ± 0·6 |
| MCP | Carcinoma | 52·6 | 3·83 ± 0·7 | 1·50 ± 0·5 | 2·55 | 6·93 ± 0·4 | 21·21 ± 0·9 |
| AME | | 121·3 | 4·47 ± 0·3 | 3·27 ± 0·7 | 1·37 | 6·58 ± 0·8 | 18·36 ± 0·5 |
| BAS | | 23·5 | 3·72 ± 0·2 | 1·29 ± 0·2 | 2·88 | 5·82 ± 0·8 | 18·67 ± 0·9 |
| HOR | | 84·1 | 9·27 ± 2·8 | 5·79 ± 2·0 | 1·60 | 7·51 ± 0·4 | 15·09 ± 0·7 |
| MCF | | 28·3 | 4·07 ± 0·5 | 2·44 ± 0·2 | 1·67 | 6·41 ± 0·3 | 18·25 ± 0·8 |
| *P* (Mann–Whitney test) | | | 0·016 | N.S. | 0·016 | N.S. | 0·028 |

N.S.: Not significant.

carcinomas as well as in the non-malignant conditions.

To summarize, the experiments described here indicate a reduced expression of the 265K and an increased expression of the 63K component on the surface of cells from carcinomas. It is suggested that these changes could be an indicator of the malignancy of the carcinomas. Further work designed to characterize these components is in progress.

This work was supported by grants from the North of England Cancer Research Campaign and the Manpower Services Commission. The authors thank Professor I. D. A. Johnston of the Department of Surgery, University of Newcastle upon Tyne, for providing tumour samples, and Dr P. A. Riley of University College School of Medicine, London, for some of the cell cultures.

### REFERENCES

BOWEN, J. G. & KULATILAKE, A. E. (1979) A common marker for cancer cells? *Br. J. Cancer*, **40**, 806.

BRAMWELL, M. E. & HARRIS, H. (1978) An abnormal membrane glycoprotein associated with malignancy in a wide range of different tumours. *Proc. R. Soc. B.*, **201**, 87.

BRUNSON, K. W., BEATTIE, G. B. & NICOLSON, G. L. (1978) Selection and altered properties of brain-colonising metastatic melanoma. *Nature*, **272**, 543.

CAMPBELL, R. C. (1967) *Statistics for Biologists.* London: Cambridge University Press.

COVE, D. H., WOODS, K. L., SMITH, S. C. & 4 others (1979) Tumour markers in breast cancer. *Br. J. Cancer*, **40**, 710.

GUY, D., LATNER, A. L. & TURNER, G. A. (1977) Radioiodination studies of tumour cell surface proteins after different disaggregation procedures. *Br. J. Cancer*, **36**, 166.

HYNES, R. O. (1973) Alteration of cell surface proteins by viral transformation and by proteolysis. *Proc. Natl Acad. Sci. U.S.A.*, **70**, 3170.

HYNES, R. O. (1979) Proteins and glycoproteins. In *Surface of Normal and Malignant Cells.* Ed. Hynes. London: John Wiley. p. 103.

PRICE, M. R. & STODDART, R. W. (1976) A characteristic protein of the surfaces of neoplastic cells. *Biochem. Soc. Trans.*, **4**, 673.

SHERBET, G. V. (1978) *The Biophysical Characterisation of the Cell Surface.* London: Academic Press. p. 136.

SHERBET, G. V. & LAKSHMI, M. S. (1974) The surface properties of some human intracranial tumour cell lines in relation to their malignancy. *Oncology*, **29**, 335.

TORMEY, D. C., WAALKES, T. P. & SIMON, R. M. (1976) Biological markers in breast carcinoma. II. Clinical correlations with human chorionic gonadotropin. *Cancer*, **39**, 2391.

TORMEY, D. C., WAALKES, T. P., SNYDER, J. J. & SIMON, R. M. (1977) Biological markers in breast carcinoma. III. Clinical correlations with carcinoembryonic antigen. *Cancer*, **39**, 2397.

TURNER, G. A., GUY, D., LATNER, A. L. & SHERBET, G. V. (1981) Cell surface changes associated with the selection of spontaneous metastases. In *Proc. EORTC Conference on Metastasis.* (In press.)

WANG, D. Y., BULBROOK, R. D., HAYWARD, J. L., HENDRICK, J. C. & FRANCHIMONT, P. (1975) Relationship between plasma carcinoembryonic antigen and prognosis in women with breast cancer. *Eur. J. Cancer*, **11**, 615.

YOGEESWARAN, G., STEIN, G. S. & SEBASTIAN, H. (1978) Altered cell surface organization of gangliosides and sialoglycoproteins of mouse metastatic melanoma variant lines selected *in vivo* for enhanced lung implantation. *Cancer Res.*, **38**, 1336


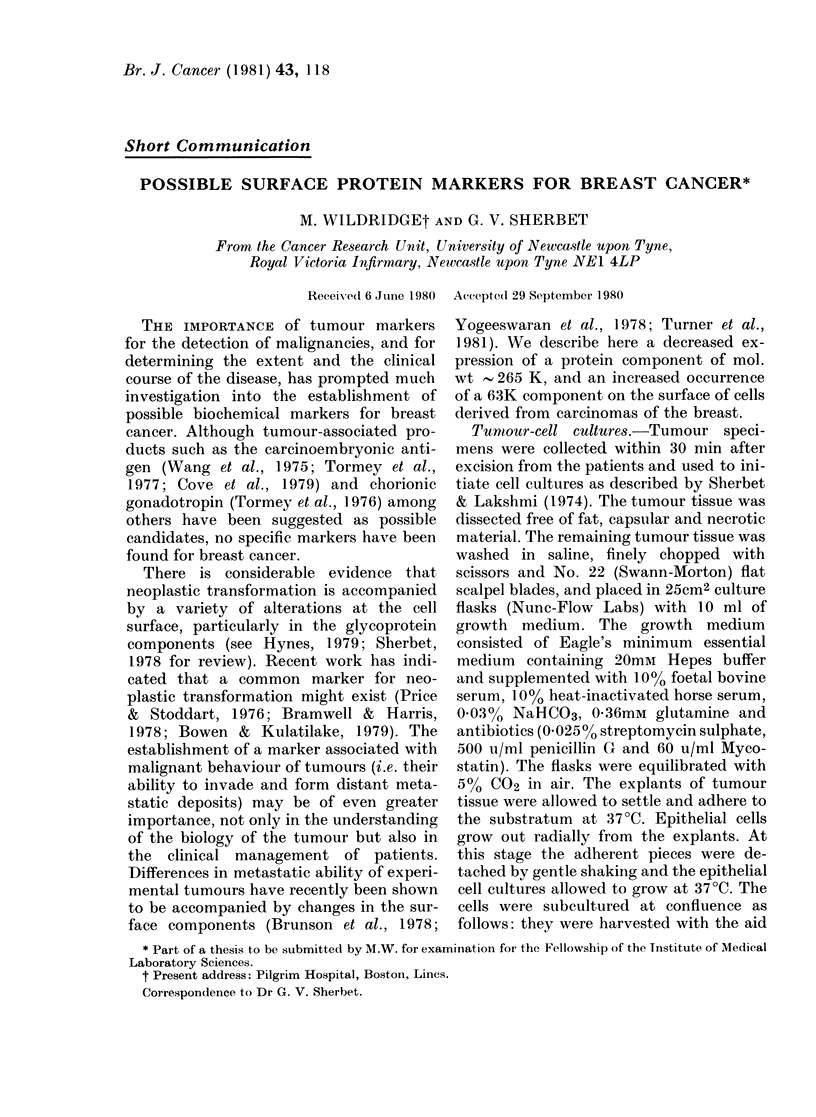

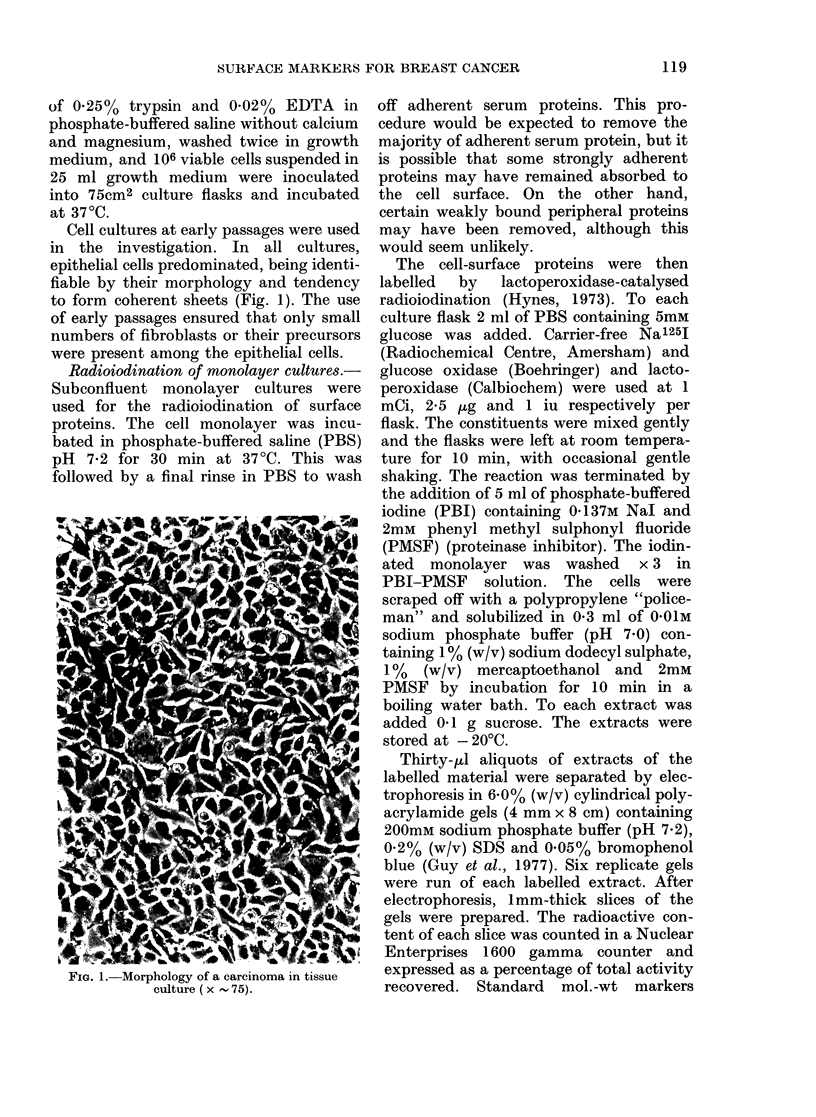

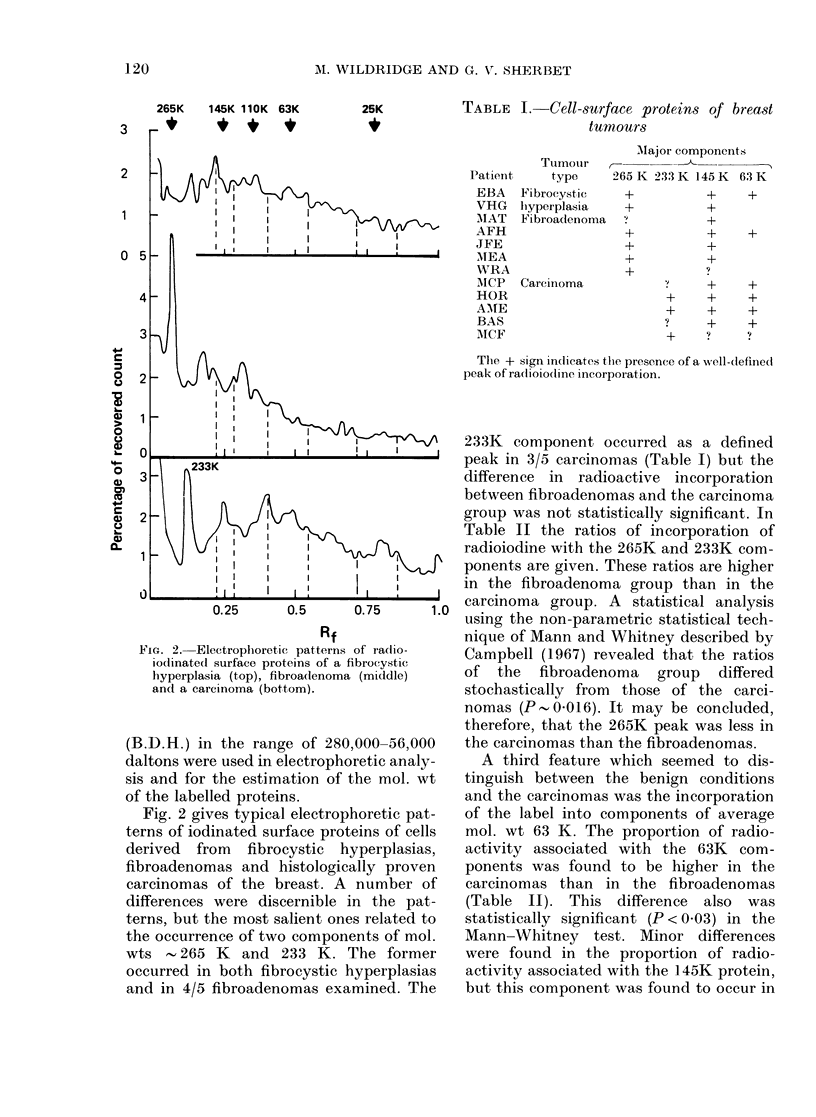

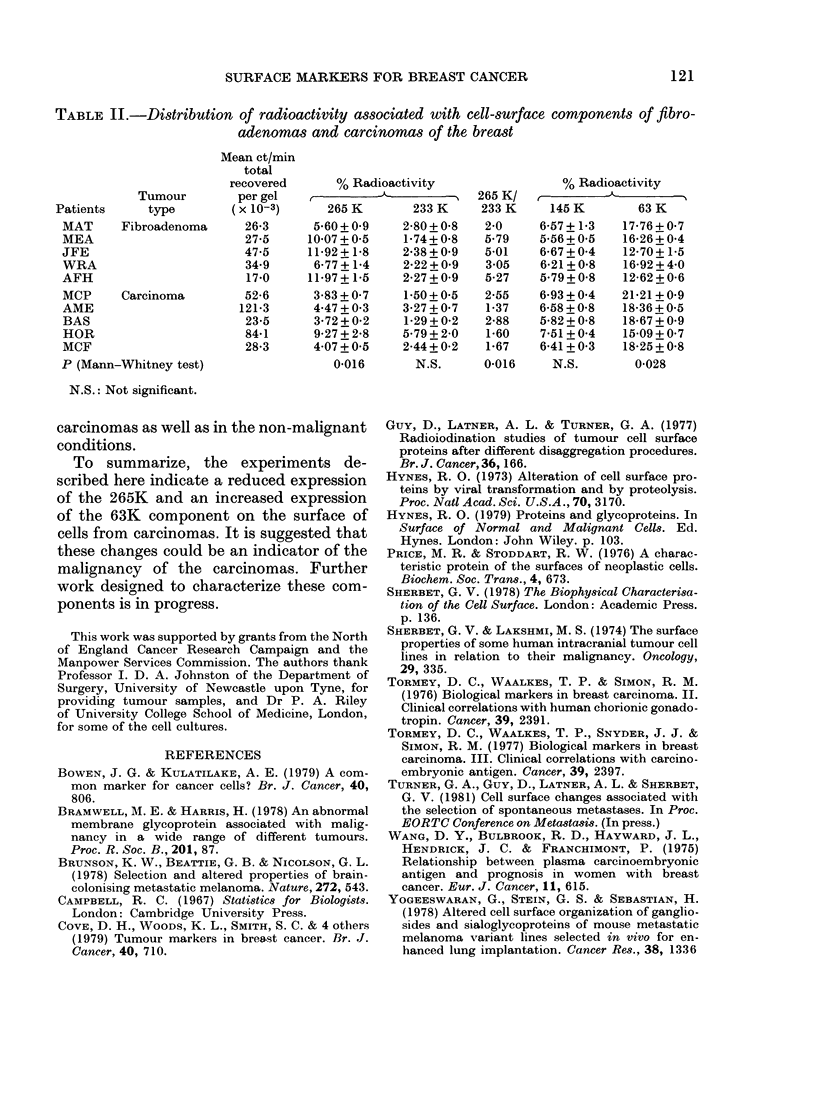


## References

[OCR_00385] Bowen J. G., Kulatilake A. E. (1979). A common marker for cancer cells?. Br J Cancer.

[OCR_00390] Bramwell M. E., Harris H. (1978). An abnormal membrane glycoprotein associated with malignancy in a wide range of different tumours.. Proc R Soc Lond B Biol Sci.

[OCR_00396] Brunson K. W., Beattie G., Nicolsin G. L. (1978). Selection and altered properties of brain-colonising metastatic melanoma.. Nature.

[OCR_00404] Cove D. H., Woods K. L., Smith S. C., Burnett D., Leonard J., Grieve R. J., Howell A. (1979). Tumour markers in breast cancer.. Br J Cancer.

[OCR_00409] Guy D., Latner A. L., Turner G. A. (1977). Radioiodination studies of tumour cell-surface proteins after different disaggregation procedures.. Br J Cancer.

[OCR_00415] Hynes R. O. (1973). Alteration of cell-surface proteins by viral transformation and by proteolysis.. Proc Natl Acad Sci U S A.

[OCR_00425] Price M. R., Stoddart R. W. (1976). A characteristic protein of the surfaces of neoplastic cells.. Biochem Soc Trans.

[OCR_00435] Sherbet G. V., Lakshmi M. S. (1974). The surface properties of some human intracranial tumour cell lines in relation to their malignancy.. Oncology.

[OCR_00441] Tormey D. C., Waalkes T. P., Simon R. M. (1977). Biological markers in breast carcinoma. II. Clinical correlations with human chorionic gonadotrophin.. Cancer.

[OCR_00447] Tormey D. C., Waalkes T. P., Snyder J. J., Simon R. M. (1977). Biological markers in breast carcinoma. III. Clinical correlations with carcinoembryonic antigen.. Cancer.

[OCR_00459] Wang D. Y., Bulbrook R. A., Hayward J. L., Hendrick J. C., Franchimont P. (1975). Relationship between plasma carcinoembryonic antigen and prognosis in women with breast cancer.. Eur J Cancer.

[OCR_00466] Yogeeswaran G., Stein B. S., Sebastian H. (1978). Altered cell surface organization of gangliosides and sialylglycoproteins of mouse metastatic melanoma variant lines selected in vivo for enhanced lung implantation.. Cancer Res.

